# Medicine of Long Ago

**Published:** 1911-05

**Authors:** 


### Medicine of Long Ago

In another department is reported a meeting of the Schuyler County Medical Society which is of unusual interest because it throws strong light upon the methods of medical practice of half a century ago. The papers read at this meeting by Dr. Clawson and Dr. Smith are illuminating to the practitioner of today and reveal in striking fashion the difficulties and the darkness in which the men of that day worked.

Dr. Clawson gives us a delightfully reminiscent picture of the manner of instruction of medical students of fifty years ago and draws graphic portraits of those giants among the medical pioneers who laid the foundations for the medical department of the University of Buffalo of today.

The paper by Dr. Smith is particularly recommended to the reading of young practitioners that they may be encouraged to more earnest effort and a calmer mind in their battles with disease conditions. Fifty years ago when bacteriology was a dream; when asepsis was practically unknown; when appendicitis was unheard of; when human intelligence and common sense took the place of the finer diagnostic methods which are now placed at our disposal while yet we are students, the physicians, one of whom tells in thrilling passages of his personal experiences, met and combatted conditions which they knew little of—and sometimes absolutely nothing—and dragged their patients from the grave. It has taken half a century to refine the methods of these early practitioners. Could anything be more classic in its description than Dr. Smith's care of an appendical abscess or of his treatment of that desperately ill pneumonia patient whom he saved?

Interesting as are these illustrative cases there is a deeper lesson to be learned from the reading of this paper. In the case of ruptured uterus which he reports, Dr. Smith when pressed for a reason for the patient's death very frankly stated that he did not know. It took great courage to say "I do not know," and well deserved were the congratulations he received from Dr. James P. White, who added that there would be fewer blunders "did we not profess to a knowledge which we do not possess."

